# IL-1β processing in mechanical ventilation-induced inflammation is dependent on neutrophil factors rather than caspase-1

**DOI:** 10.1186/2197-425X-1-8

**Published:** 2013-10-29

**Authors:** Kim Timmermans, Selina EI van der Wal, Michiel Vaneker, Jeroen AWM van der Laak, Mihai G Netea, Peter Pickkers, Gert Jan Scheffer, Leo AB Joosten, Matthijs Kox

**Affiliations:** Department of Anaesthesiology, RUNMC, Nijmegen, 6500 HB The Netherlands; Department of Pathology, RUNMC, Nijmegen, 6500 HB The Netherlands; Department of Intensive Care Medicine, RUNMC, Internal mail 710, P.O. Box 9101, Nijmegen, 6500 HB The Netherlands; Department of Internal Medicine, RUNMC, Nijmegen, 6500 HB The Netherlands

**Keywords:** VILI, Inflammation, Ventilation, IL-1β

## Abstract

**Purpose:**

Mechanical ventilation can cause ventilator-induced lung injury, characterized by a sterile inflammatory response in the lungs resulting in tissue damage and respiratory failure. The cytokine interleukin-1β (IL-1β) is thought to play an important role in the pathogenesis of ventilator-induced lung injury. Cleavage of the inactive precursor pro-IL-1β to form bioactive IL-1β is mediated by several types of proteases, of which caspase-1, activated within the inflammasome, is the most important. Herein, we studied the roles of IL-1β, caspase-1 and neutrophil factors in the mechanical ventilation-induced inflammatory response in mice.

**Methods:**

Untreated wild-type mice, IL-1αβ knockout and caspase-1 knockout mice, pralnacasan (a selective caspase-1 inhibitor)-treated mice, anti-keratinocyte-derived chemokine (KC)-treated mice and cyclophosphamide-treated neutrophil-depleted wild-type mice were ventilated using clinically relevant ventilator settings (tidal volume 8 ml/kg). The lungs and plasma were collected to determine blood gas values, cytokine profiles and neutrophil influx.

**Results:**

Mechanical ventilation resulted in increased pulmonary concentrations of IL-1β and KC and increased pulmonary neutrophil influx compared with non-ventilated mice. Ventilated IL-1αβ knockout mice did not demonstrate this increase in cytokines. No significant differences were observed between wild-type and caspase-1-deficient or pralnacasan-treated mice. In contrast, in anti-KC antibody-treated mice and neutropenic mice, inflammatory parameters decreased in comparison with ventilated non-treated mice.

**Conclusions:**

Our results illustrate that IL-1 is indeed an important cytokine in the inflammatory cascade induced by mechanical ventilation. However, the inflammasome/caspase-1 appears not to be involved in IL-1β processing in this type of inflammatory response. The attenuated inflammatory response observed in ventilated anti-KC-treated and neutropenic mice suggests that IL-1β processing in mechanical ventilation-induced inflammation is mainly mediated by neutrophil factors.

**Electronic supplementary material:**

The online version of this article (doi:10.1186/2197-425X-1-8) contains supplementary material, which is available to authorized users.

## Introduction

Mechanical ventilation is a life-saving therapy, although it can also cause ventilator-induced lung injury (VILI) [1]. VILI is characterized by a sterile inflammatory response in the lungs resulting in tissue damage that may sustain respiratory failure. The mechanical ventilation-induced inflammatory response can also spread systemically, which in severe cases can result in multi-organ dysfunction syndrome (MODS) [2]. Even protective ventilation strategies that do not cause direct mechano-induced tissue damage (baro- or volutrauma) have been shown to elicit the release of pro-inflammatory cytokines, recruitment of leukocytes and subsequent lung injury [3, 4]. The mechanisms behind this so-called 'biotrauma’ have not yet been completely elucidated.

Previous studies have demonstrated that the TLR4/TRIF pathway is important in the mechanical ventilation-induced inflammatory response [4, 5]. Furthermore, it is becoming increasingly clear that the pro-inflammatory cytokine interleukin-1β (IL-1β) plays a key role in the pathogenesis of the inflammatory response and VILI by promoting neutrophil recruitment and by increasing epithelial injury and permeability [6–8]. Through recognition by the IL-1 receptor (IL-1R), not only the secreted IL-1β but also the cell-associated family member IL-1α may stimulate production of other inflammatory cytokines via IL-1R-associated kinases (IRAKs) and thereby positively amplify the inflammatory response [9]. However, up till now, this has not been studied in the context of mechanical ventilation-induced inflammation.

Upon activation of the innate immune system, e.g. via TLRs, IL-1β is synthesized as an inactive precursor molecule, pro-IL-1β, that cannot bind and activate the IL-1R [10]. In order to process pro-IL-1β and form bioactive IL-1β, proteolytic cleavage of the N-terminal 116 amino acids from the precursor is required. Caspase-1 is the major protein implicated in cleavage of pro-IL-1β [10, 11].

Caspase-1 exists as an inactive zymogen in cells of myeloid origin (e.g. tissue macrophages, dendritic cells) which needs to be activated to perform its proteolytic tasks [9]. Caspase-1 is also known to be expressed in a wide range of other cell types including lung fibroblasts and epithelial cells [12, 13]. The inflammasome is a protein platform that is responsible for the activation of caspase-1 [10, 14]. A broad range of infectious and autoimmune diseases that involve IL-1β have been associated with inappropriate activation of the inflammasome [12, 14, 15], while in several other disease models in which IL-1β plays a crucial role, the inflammasome appears not to be involved [16, 17]. IL-1β processing in these models might rely on neutrophil serine proteases, like elastase, granzyme A, cathepsin G or proteinase-3 [10, 18–20]. Hitherto, the role of caspase-1 in processing of IL-1β in the mechanical ventilation-induced inflammatory response is unknown.

We studied the roles of IL-1β, caspase-1 and neutrophil factors in the mechanical ventilation-induced inflammatory response in mice ventilated with clinically relevant ventilator settings.

## Materials and methods

All experiments were approved by the Regional Animal Ethics Committee in Nijmegen and performed under the guidelines of the Dutch Council for Animal Care and the National Institutes of Health. They have therefore been performed in accordance with the ethical standards laid down in the 1964 Declaration of Helsinki and its later amendments.

### Animals

Age-matched wild-type C57Bl/6 mice and extensively backcrossed caspase-1 knockout mice or IL-1αβ knockout mice (aged 8 to 14 weeks, weight 25 ± 4 g) with C57Bl/6 background were used in this study. The mice were housed in a light- and temperature-controlled room under specific pathogen-free (SPF) conditions. Standard pelleted chow (1.00% Ca, 0.22% Mg, 0.24% Na, 0.70% P, 1.02% K, SSNIFF Spezialdiäten GmbH, Soest, Germany) and drinking water were available *ad libitum*. These conditions are similar to previous studies in which this mouse model was used [4, 5, 21, 22].

### Experimental design

#### IL-1αβ knockout experiments

IL-1 can induce inflammation via activation of the IL-1 receptor. To study whether IL-1 is indeed involved in initiation and/or propagation of the inflammatory cascade induced by mechanical ventilation, mechanically ventilated IL-1αβ^-^/_-_ mice (*n* = 8) were compared with ventilated wild-type mice (*n* = 8). As controls, non-ventilated IL-1αβ^-^/_-_ (*n* = 8) and wild-type mice (*n* = 8) were used.

#### Caspase-1 experiments

Caspase-1 is able to cleave the inactive precursor pro-IL-1β to form the active cytokine IL-1β. To study the role of caspase-1 in the mechanical ventilation-induced inflammatory response, mechanically ventilated caspase-1 knockout mice (*n* = 8) and ventilated wild-type mice treated with the selective caspase-1 inhibitor pralnacasan (100 mg/kg) (*n* = 8) were compared with ventilated untreated wild-type mice (*n* = 8) [23, 24]. As controls, non-ventilated caspase-1^-^/_-_, pralnacasan-treated wild-type and untreated wild-type mice (*n* = 8 per group) were used.

#### Anti-KC antibody experiments

Apart from caspase-1, neutrophil serine proteases are also able to process IL-1β [8]. In order to investigate whether the attraction of neutrophils by the chemo-attractant keratinocyte-derived chemokine (KC) is involved in the inflammatory response elicited by mechanical ventilation, mechanically ventilated wild-type mice treated with an intraperitoneal dose of 100 μg of a neutralizing monoclonal anti-KC antibody (R&D Systems, Minneapolis, MN, USA) 1 h before induction of anaesthesia (*n* = 8) were compared with ventilated untreated wild-type mice (*n* = 8). As controls, non-ventilated untreated wild-type mice (*n* = 8) were used.

#### Neutrophil depletion experiments

Neutrophil serine proteases are able to process IL-1β [8]. In order to study the possible role of neutrophil factors in IL-1β processing in the mechanical ventilation-induced inflammatory response, mechanically ventilated neutrophil-depleted wild-type mice (*n* = 8) were compared with ventilated untreated wild-type mice (*n* = 8). As controls, non-ventilated wild-type mice (*n* = 8) were used. The neutrophil-depleted group was neutrophil-depleted with cyclophosphamide as described previously [25, 26].

### Experimental procedures

The mice were anaesthetized using an intraperitoneal injection of 7.5 μl per gram body weight of KMA mix (25.5 mg/ml ketamine, 40 μg/ml medetomidine, 0.1 mg/ml atropine in saline). Subsequently, the animals were orally intubated, an arterial line was placed in the arteria carotis, and the mice were mechanically ventilated (MiniVent*®*, Hugo Sachs Elektronik-Harvard Apparatus, March-Hugstetten, Germany). The ventilation settings used were based on measured tidal volume and respiratory rate during spontaneous ventilation in C57Bl/6 mice [27]: a tidal volume of 8 ml/kg body weight and a frequency of 150/min. All animals received 4 cm H_2_O positive end-expiratory pressure (PEEP), and fraction of inspired oxygen was set to 0.4. In order to maintain anaesthesia, boluses of 5.0 μl per gram body weight maintenance KMA mix (3.6 mg/ml ketamine, 4 μg/ml medetomidine, 7.5 μg/ml atropine in saline) were given every 30 min via an intraperitoneally placed catheter. Rectal temperature was monitored continuously and maintained between 36.0°C and 37.5°C using a heating pad. After the 4-h ventilation period, the mice were sacrificed by exsanguination under anaesthesia. The control mice were anaesthetized, but not ventilated, and sacrificed shortly after induction of anaesthesia. Tissue and blood were sampled in order to determine blood gas values (only in ventilated mice), cytokine production and neutrophil influx.

Lipopolysaccharide (LPS) was measured in the mechanical ventilation circuit by Limulus Amebocyte Lysate testing (Cambrex Bio Science, Walkersville, MD, USA; detection limit: 0.06 IU/ml) to rule out contamination and LPS-induced pulmonary inflammation.

### Tissue harvesting

Plasma was isolated by centrifugation at 13,000*g* for 5 min and stored at –80°C. Immediately after exsanguination, the heart and lungs were carefully removed *en block* via midline sternotomy. The right middle lung lobe was fixed in 4% buffered formalin solution overnight at room temperature. The right lung was snap-frozen in liquid nitrogen and stored at –80°C. The left lung was snap-frozen and placed in 500 μl lysis buffer containing PBS, 0.5% Triton X-100 and protease inhibitor (complete EDTA-free tablets, Roche, Woerden, The Netherlands). Subsequently, the lungs were homogenized using a polytron and subjected to two rapid freeze-thaw cycles using liquid nitrogen. Finally, homogenates were centrifuged (10 min, 16,000*g*, 4°C), and the supernatant was stored at -80°C until further analysis.

### Pulmonary neutrophil influx

After overnight incubation in 4% buffered formalin solution, the right middle lung lobe was dehydrated and embedded in paraplast (Amstelstad, Amsterdam, The Netherlands). Sections of 4-μm thickness were used. Enzyme histochemistry using chloracetatesterase (LEDER staining) was used to visualize the enzyme activity in the neutrophils. Neutrophils were counted manually (ten fields per mouse), and after automated correction for air/tissue ratio, the average number of neutrophils per square centimetre per mouse was calculated.

### Biochemical analysis

**Figure 1:**
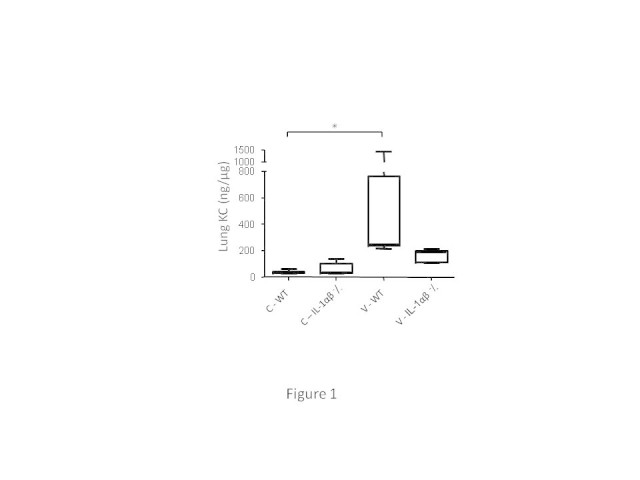
**Involvement of IL-1 in the mechanical ventilation-induced inflammatory response.** KC levels in lung homogenates expressed as nanogram cytokine per microgram total protein. Data are expressed as box-and-whiskers plots, with min to max range as whiskers. Results of analysis in the non-ventilated (C) and ventilated (V) wild-type (WT) mice and IL-1αβ knockout (^-^/_-_) mice are shown. *p < 0.05.

**Figure 2:**
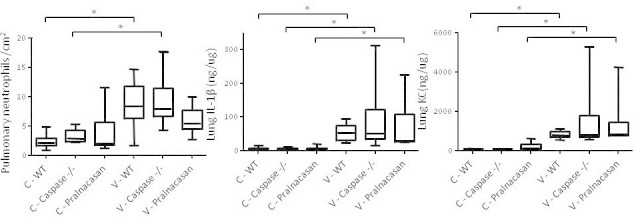
**Involvement of caspase-1 in the mechanical ventilation-induced inflammatory response.** Pulmonary neutrophil counts expressed as the number of neutrophils per square centimetre tissue and IL-1β and KC levels in lung homogenates expressed as nanogram cytokine per microgram total protein. Data are expressed as box-and-whiskers plots, with min to max range as whiskers. Results of analysis in the non-ventilated (C) and ventilated (V) wild-type (WT) mice, caspase-1 knockout (^-^/_-_) mice and pralnacasan-treated mice are shown. *p < 0.05.

**Figure 3:**
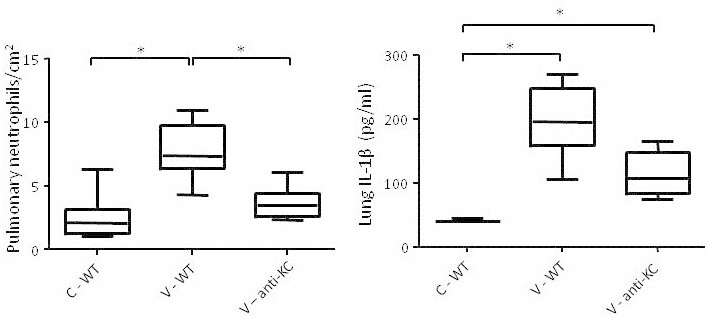
**Involvement of KC in the mechanical ventilation-induced inflammatory response.** Pulmonary neutrophil counts expressed as the number of neutrophils per square centimetre tissue and IL-1β concentration expressed as picogram cytokine per millilitre lung homogenate. Data are expressed as box-and-whiskers plots, with min to max range as whiskers. Pulmonary neutrophils and IL-1β concentration in the non-ventilated (C) and ventilated (V) untreated wild-type mice (WT) and anti-KC antibody-treated wild-type (anti-KC) mice are shown. *p < 0.05.

**Figure 4:**
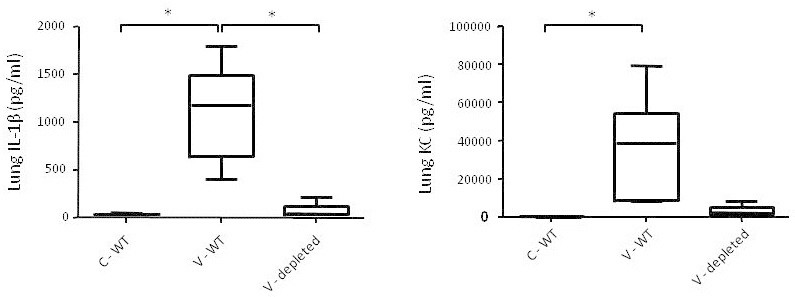
**Effects of neutrophil depletion on the mechanical ventilation-induced inflammatory response.** IL-1β and KC concentrations expressed as picogram cytokine per millilitre lung homogenate, measured in the non-ventilated (C) and ventilated (V) untreated wild-type (WT) and cyclophosphamide-treated neutrophil-depleted mice. Data are expressed as box-and-whiskers plots, with min to max range as whiskers. *p < 0.05.

KC (murine equivalent of human IL-8) in the lung homogenate was determined by enzyme-linked immunosorbent assay (ELISA; R&D Systems, Minneapolis, MN, USA). The lower detection limit is 160 pg/ml. IL-1β in the lung homogenate was determined using a radioimmunoassay (RIA) as described previously [28]. In the samples of the IL-1αβ (Figure 1) and caspase (Figure 2) experiments, total protein concentrations in the lung homogenates were determined using a BCA protein assay (Thermo Fisher Scientific, Etten-Leur, The Netherlands), and cytokine concentrations in the homogenates were normalized for protein concentration and therefore expressed as nanogram cytokine per microgram protein. In the anti-KC (Figure 3) and neutrophil depletion (Figure 4) experiments, cytokine concentrations in the lung homogenate were not normalized for total protein content due to insufficient sample volume and therefore expressed as picogram per millilitre.

### Statistical analysis

Data were not normally distributed (determined using the Kolmogorov-Smirnov and Shapiro-Wilk tests) and therefore expressed as median and range or median and interquartile range (IQR). Differences between groups were analyzed using the Kruskal-Wallis and Dunn's *post hoc* tests. Statistical analysis was performed using GraphPad Prism 5 software (GraphPad Software, La Jolla, CA, USA). *P* values <0.05 were considered significant.

## Results

Mean arterial pressure remained stable and above 65 mmHg in all animals throughout the mechanical ventilation period. Blood gas values that were obtained at the end of the ventilation period did not indicate substantial lung injury (Table 1).

**Table 1:** **Blood gas values after 4 h of ventilation**

	Median	IQR
pH	7.36	7.25 to 7.38
pCO_2_	4.73	4.17 to 5.18
PO_2_	15.3	14.6 to 17.5
BE	-5.5	-7.3 to –4.0
HCO_3_	20.2	18.3 to 20.7
TCO_2_	21.0	19.8 to 21.5
sO_2_%	99%	98 to 99
Lac	0.98	0.90 to 1.16

Values (median and IQR) from a representative ventilated group (wild-type ventilated mice used as the control group for caspase-1^-^/_-_ and pralnacasan-treated mice).

### Involvement of IL-1 in the mechanical ventilation-induced inflammatory response

After 4 h of mechanical ventilation, pulmonary levels of pro-inflammatory cytokine KC significantly increased in wild-type mice compared with non-ventilated wild-type mice. In contrast, ventilated IL-1αβ knockout mice did not show an increase in pulmonary cytokines compared with non-ventilated IL-1αβ knockout mice (Figure 1).

### Involvement of caspase-1 in the mechanical ventilation-induced inflammatory response

Pulmonary neutrophil influx significantly increased in mechanically ventilated mice compared with non-ventilated wild-type and caspase-1^-^/_-_ mice, but no differences were observed between wild-type mice, caspase-1^-^/_-_ mice or pralnacasan-treated mice. Similar to the results described above, 4 h of mechanical ventilation resulted in increased IL-1β and KC concentrations in lung homogenates in all groups. However, no significant differences in lung cytokine levels were observed between wild-type mice, caspase-1^-^/_-_ mice or pralnacasan-treated mice. (Figure 2)

### Involvement of neutrophil factors in the mechanical ventilation-induced inflammatory response

To determine whether neutrophil factors are involved in the mechanical ventilation-induced inflammatory response and IL-1β processing, we investigated the effects of treatment with an antibody against KC. KC is one of the major factors involved in neutrophil attraction to the site of inflammation (chemo-attractants). Mechanical ventilation resulted in increased levels of pulmonary neutrophils (Figure 3). This increase was abrogated by pre-treatment with an anti-KC antibody. Furthermore, the mechanical ventilation-induced increase in pulmonary IL-1β levels was less pronounced in anti-KC-treated mice compared with untreated mice, although this did not reach statistical significance (Figure 3).

To further confirm the role of neutrophil factors, we investigated the effects of mechanical ventilation following neutrophil depletion using cyclophosphamide. The effect of cyclophosphamide was visually inspected, and no pulmonary neutrophils were present (data not shown). As depicted in Figure 4, the mechanical ventilation-induced increase in pulmonary IL-1β and KC concentrations was diminished in neutrophil-depleted mice.

**Figure 5:**
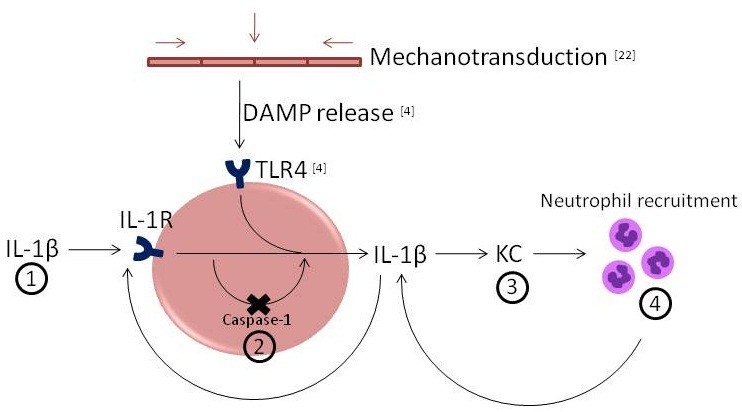
**Hypothesis regarding the role of IL-1β processing in the inflammatory response following mechanical ventilation.** We present the following hypothesis based on our results and previous findings. Mechanical ventilation causes mechanotransduction and cell and/or tissue damage. This causes the release of danger-associated molecular patterns (DAMPs) that activate TLR4 and possibly other pattern recognition receptors. Ligation of these receptors induces production of cytokines, most importantly IL-1β. Subsequently, KC is produced, leading to neutrophil recruitment to the lungs. Pro-IL-1β processing to bioactive IL-1β could occur intracellularly by caspase-1, although in our model, it only plays a minor role in IL-1β bioactivation, not excluding that it may be involved at the onset of the inflammatory process, when very few neutrophils are present. The majority of pro-IL-1β is excreted in the inactive form and then cleaved by factors released by neutrophils, such as neutrophil serine proteases. Finally, active IL-1β present extracellularly binds to the IL-1R, which in turn leads to the production of more cytokines and hence positive amplification of the inflammatory response. As such, a positive feedback loop is activated which could be an explanation for the extensive inflammatory response observed following mechanical ventilation. Numbers 1 to 4 represent the experiments performed in this study and correspond to the figure numbers in this paper. References [4] and [22] refer to previous studies performed by our group.

Our hypothesis regarding the role of IL-1β processing in the inflammatory response following mechanical ventilation is illustrated in Figure 5.

## Discussion

Consistent with previous results published by our group [4, 5, 22] and others [29, 30], the present study shows that mechanical ventilation using clinically relevant settings induces a pulmonary inflammatory response in mice. In addition, our data is in support of previous findings that IL-1 plays an important role in initiation and/or propagation of the mechanical ventilation-induced inflammatory response and suggests that processing of IL-1β in mechanical ventilation-induced inflammation occurs via the release of neutrophil factors and not through caspase-1-dependent mechanisms.

Our finding that caspase-1 does not play a significant role in mechanical ventilation-induced inflammation is in contrast to a recent study where the NLRP3 inflammasome was found to play an important role in the mechanical ventilation-induced inflammatory response and VILI [31]. Several differences between their study and ours might explain the different results. First, in the previous study, ASC and NLRP3 (components of the inflammasome upstream of caspase-1) knockout mice were used, and it was shown that mechanical ventilation activated caspase-1 in a NLRP3-dependent fashion. Nevertheless, it is very well possible that ASC and NLRP3 play other roles in the mechanical ventilation-induced inflammatory cascade than merely activating caspase-1. As abrogation and inhibition of caspase-1 by either a knockout approach or pralnacasan treatment did not have any effect in our model, the role of caspase-1/the inflammasome appears not to be as crucial as suggested. Second, differences between wild-type and ASC or NLRP3 knockout were only found at a high tidal volume of 15 ml/kg, known to cause extensive lung damage [22], while no effects were found at a low tidal volume of 7.5 ml/kg, which is more representative of the current clinical practice and similar to that used in the present study. This suggests that the inflammasome might play a more important role at higher tidal volumes which lead to apparent lung injury but not in mechanical ventilation-induced inflammation at clinically relevant ventilator settings. Interestingly, a more recent study from the same group showed that pre-treatment with allopurinol or uricase (both degraders of known inflammasome-activating factors [32]) did not decrease mechanical ventilation-induced inflammation, which is in support of a caspase/inflammasome-independent mechanism [33]. As beneficial effects of uricase and allopurinol were observed in terms of alveolar barrier dysfunction, it appears plausible that ASC and NLRP3 are involved in VILI via inflammation-independent mechanisms.

The pronounced influx of neutrophils in the lung observed in our experiments suggests a major role for these inflammatory cells in the inflammatory cascade following mechanical ventilation. Our findings that treatment with an antibody against KC or depletion of neutrophils reduced the mechanical ventilation-induced production of IL-1β and KC indicate an important role for neutrophils in initiation and/or propagation of the inflammatory response. In this respect, pro-IL-1β cleavage in our model is probably achieved through neutrophil factors, such as the serine proteases proteinase-3 (PR-3), elastase or cathepsin G, leading to bioactive IL-1β and propagation of the inflammatory response through binding of the IL-1-receptor, which in turn leads to production of other inflammatory cytokines such as KC [10, 34, 35]. Several other IL-1β-mediated inflammatory responses are described to be partly or completely independent of the inflammasome and caspase-1 and possibly dependent on neutrophil factors, including proteinase-3 and cathepsin G [35]. Future studies should focus on the confirmation of our hypothesis and the identification of these neutrophil factors.

Our study has several limitations. First, we used cyclophosphamide to deplete neutrophils. While this is a widely used method [25, 26, 36, 37], cyclophosphamide treatment may also result in depletion of other cell types that play a role in mechanical ventilation-induced inflammation [38, 39]. Nevertheless, our data of mice treated with an anti-KC antibody underline the importance of neutrophils in this process. Second, no histological slides to perform neutrophil counts were collected in the IL-1αβ^-^/_-_ experiments to investigate whether these knockout mice were still able to recruit neutrophils. Finally, we cannot exclude the possibility that next to mechanical ventilation, the procedures related to the instrumentation/ventilation (e.g. intubation, arterial cannulation) also induce inflammation to a certain extent. However, we have previously shown that the inflammatory response is aggravated when mice are ventilated with these parameters for a longer period of time or when higher tidal volumes are used, suggesting that the inflammatory response is mainly ventilation-induced.

## Conclusions

In conclusion, our results indicate that IL-1 signalling is important in mechanical ventilation-induced inflammation. We show that following mechanical ventilation, IL-1β bioactivation is not caspase-1 dependent but appears to be mediated by neutrophil factors, leading to a positive amplification loop and further propagation of the inflammatory response. Further elucidation of the precise mechanism of IL-1β processing in mechanical ventilation-induced inflammation could provide novel targets for the future treatment of VILI [40].

## Authors’ original submitted files for images

Below are the links to the authors’ original submitted files for images. Authors’ original file for figure 1 Authors’ original file for figure 2 Authors’ original file for figure 3 Authors’ original file for figure 4 Authors’ original file for figure 5
