# Supplementary figures and images for: IL-1β processing in mechanical ventilation-induced inflammation is dependent on neutrophil factors rather than caspase-1
Source: Intensive Care Med Exp. 2013 Oct 29;1:8. doi: 10.1186/2197-425X-1-8 (PMC4797957; doi:10.1186/2197-425X-1-8)

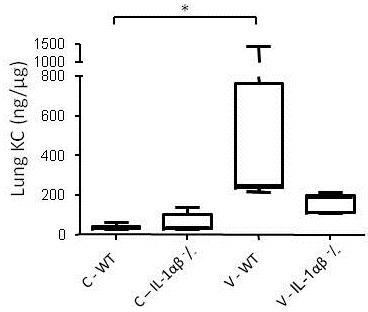

Supplement: Supplementary file 1 — Authors’ original file for figure 1 [file 40635_2013_27_MOESM1_ESM.jpeg]

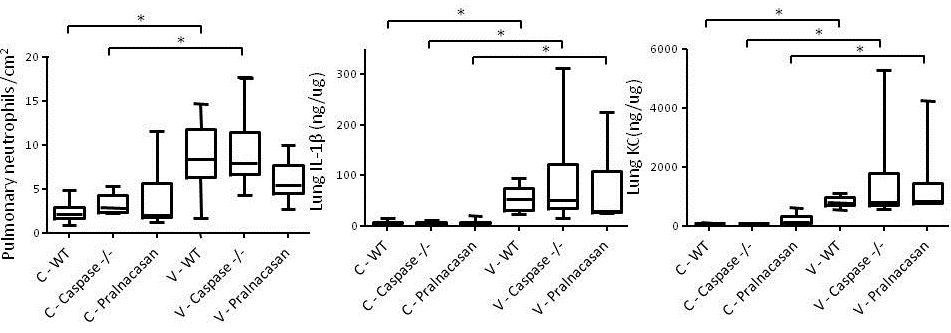

Supplement: Supplementary file 2 — Authors’ original file for figure 2 [file 40635_2013_27_MOESM2_ESM.jpeg]

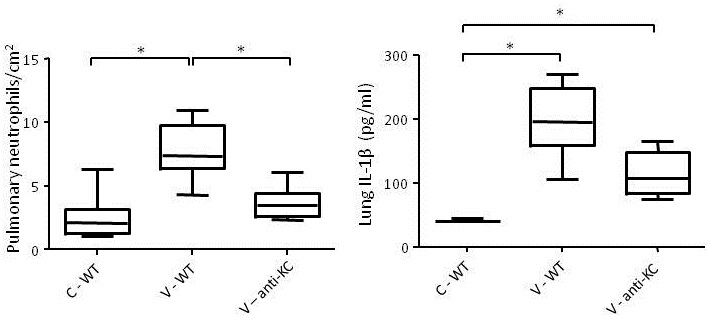

Supplement: Supplementary file 3 — Authors’ original file for figure 3 [file 40635_2013_27_MOESM3_ESM.jpeg]

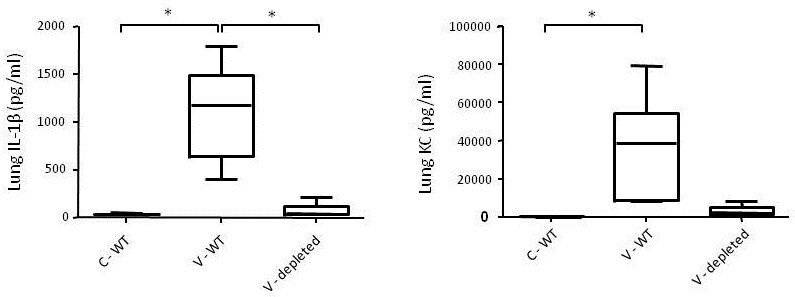

Supplement: Supplementary file 4 — Authors’ original file for figure 4 [file 40635_2013_27_MOESM4_ESM.jpeg]

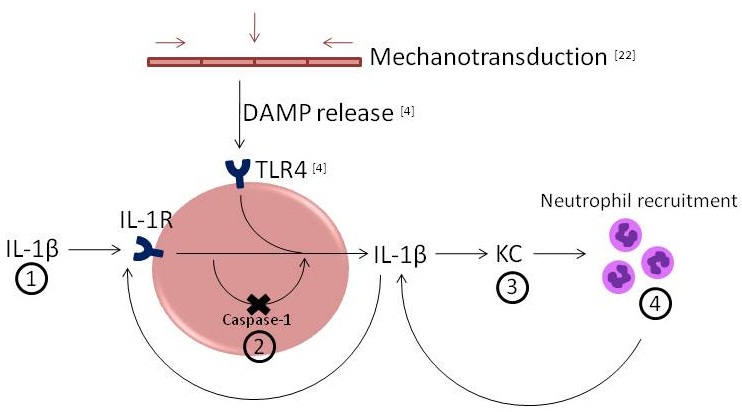

Supplement: Supplementary file 5 — Authors’ original file for figure 5 [file 40635_2013_27_MOESM5_ESM.jpeg]
